# Perioperative Anaphylaxis: A Systematic Approach to Evaluate High-Risk Drug Allergy

**DOI:** 10.1155/crcc/8845963

**Published:** 2025-04-07

**Authors:** Alexandra Navard-Keck, Stanislaw J. Gabryszewski, Emily S. Robbins, Joseph Cafone, Juhee Lee

**Affiliations:** ^1^Division of Allergy and Immunology, Children's Hospital of Philadelphia, Philadelphia, Pennsylvania, USA; ^2^National Institute of Allergy and Infectious Diseases, National Institutes of Health, Bethesda, Maryland, USA

**Keywords:** drug allergy, drug provocation testing, perioperative anaphylaxis, skin testing

## Abstract

Determining the etiology of perioperative anaphylaxis is a challenging task, as multiple medications are often administered simultaneously during anesthesia. This is compounded by the paucity of validated skin testing. While drug challenges are the definitive means of assessing for IgE-mediated drug allergy, they must be weighed with the risk of severe reaction. As such, multidisciplinary collaboration is warranted to ensure drug provocation testing is performed thoughtfully and safely. Here, we present a case of an 18-year-old male with juvenile kyphosis who underwent anesthesia prior to spinal fusion surgery. He was given intravenous fentanyl, propofol, dexamethasone, remifentanil, tranexamic acid, methadone, and cefazolin. Additionally, iodine, chlorhexidine, and tincture of benzoin were applied topically. Shortly after the start of anesthesia and prior to incision, he developed bronchospasm, hypoxia, hypotension, and pulseless electrical activity with a return of spontaneous circulation following cardiopulmonary resuscitation. A tryptase level drawn during the event was elevated at 23.7 *μ*g/L (reference range: 0–11.4 *μ*g/L). Months later, the patient underwent skin prick and intradermal testing in an allergy clinic, which was largely unrevealing for a culprit. Given the absence of validated predictive values for skin testing, drug provocation testing was performed with the patient admitted to the intensive care unit due to the high-risk nature of testing. Medications were selected for a challenge after multidisciplinary discussions with specialists in anesthesia and surgery based on the availability of alternative medications. Following negative drug provocation testing to intravenous dexamethasone, intravenous fentanyl, oral midazolam, intravenous methadone, and intravenous tranexamic acid, as well as topical challenges to chlorhexidine, iodine, and tincture of benzoin, the patient was instructed to continue to avoid cefazolin, propofol, and remifentanil and was able to subsequently undergo spinal fusion surgery safely. This case demonstrates a systematic approach for high-risk drug allergy testing that was facilitated by collaboration with allergy, intensive care, anesthesia, and surgery.

## 1. Introduction

Perioperative anaphylaxis (POA) is a rare event that occurs at a rate of approximately 15 per 100,000 surgical cases. POA is associated with burdensome healthcare costs, longer hospital lengths of stay, and a greater risk of mortality than other forms of anaphylaxis [1]. The clinical presentation of POA is unique. Cardiovascular and respiratory symptoms are the most common presenting features, and, in contrast with other forms of anaphylaxis, cutaneous symptoms are variable. Hypothesized explanations for unremarkable cutaneous symptoms include decreased circulation and masking of symptoms due to surgical draping [2]. Importantly, identifying the causal agent in POA is challenging, as patients typically receive multiple medications in a short time frame. Known causal agents include antibiotics, neuromuscular blocking agents (NMBAs), antiseptics, latex, and dyes. There is geographic variability in the most common causal agents, in part due to variation in exposure to preprocedure sensitizing factors [1]. In the United States, beta-lactam antibiotics—mainly cefazolin—are commonly implicated, and the overall incidence of latex allergy is decreasing. In some European countries, NMBAs are the most common culprit agents [3]. In this report, we present a case of POA that necessitated step-wise, high-risk drug allergy testing requiring intensive care unit (ICU)–level care. This case emphasizes the importance of collaboration among specialists within allergy, critical care, anesthesiology, and surgery in the evaluation and management of POA.

## 2. Case Presentation and Discussion

An 18-year-old male with Scheuermann's kyphosis, anxiety, depression, and no known allergies underwent anesthesia prior to spinal fusion surgery. This was his first surgery. At presentation, he was clinically well with no signs or symptoms of illness. He received multiple medications in the perioperative period. At 0806, he received oral midazolam. Between 0850 and 0900, the following medications were administered: intravenous (IV) fentanyl, IV propofol, IV dexamethasone, and IV remifentanil. He was intubated at 0854 with an endotracheal tube containing latex. Between 0930 and 0940, IV tranexamic acid and IV methadone were administered. At 1011, he received IV cefazolin. His skin was prepped and cleaned with topical iodine, chlorhexidine, and tincture of benzoin.

At 1020, the patient developed tachycardia, increased airway resistance, hypoxemia, and hypotension, which progressed to pulseless electrical activity and cardiac arrest. He received 1000 mcg of epinephrine at 1020, 1027, and 1035. He required 15 min of cardiopulmonary resuscitation, with the return of spontaneous circulation. He was started on an epinephrine infusion and was also given IV calcium, nebulized albuterol, IV dexamethasone, IV diphenhydramine, IV ketamine, IV magnesium, and IV normal saline. The tryptase level drawn 40 min after the onset of symptoms was 23.7 *μ*g/L (reference range: 0–11.4 *μ*g/L). Baseline tryptase was 3.3 *μ*g/L, and latex-specific serum IgE was negative. He was admitted to the ICU and ultimately recovered well.

The patient's history, clinical symptoms, and elevated tryptase are consistent with POA. The following agents were considered potential culprits of this event:

1. Cefazolin: Antibiotics are reported as the culprit agent in 40–55% of POA events in the United States [2].
2. Chlorhexidine: This common disinfectant is estimated to account for 9–10% of POA cases [2].
3. Dexamethasone: Glucocorticoids are a rare cause of anaphylaxis. There have been case reports of anaphylaxis to various glucocorticoids, with a rare report implicating dexamethasone [3, 4].
4. Fentanyl and remifentanil: These medications are synthetic opioids and are very rarely associated with anaphylaxis [5].
5. Methadone: To our knowledge, there are no documented cases of anaphylaxis to methadone.
6. Midazolam: Hypnotics are a rare cause of anaphylaxis [6].
7. Propofol: This sedative is an uncommon cause of anaphylaxis and is estimated to account for 1.2% of anaphylaxis cases. The implicated allergenic antigens are the isopropyl groups in propofol [7].
8. Tranexamic acid: This fibrinolytic is a rare cause of POA [8].
9. Tincture of benzoin: This antiseptic is a rare cause of POA and more commonly causes contact dermatitis [9].
10. Topical iodine: This antiseptic is a rare cause of POA [6].
11. Latex: Latex was previously a common culprit in POA. Recently, latex-associated POA rates have been declining [2].

Three months after the reaction, the patient presented to the allergy clinic for drug allergy testing to all suspected agents. Latex was ruled out as the culprit agent due to negative specific IgE and previous tolerance to latex exposures. Skin prick testing (SPT) performed over multiple outpatient clinic visits was negative for cefazolin, dexamethasone, fentanyl, methadone, midazolam, propofol, remifentanil, tranexamic acid, and chlorhexidine gluconate 2% (Table 1). Intradermal testing (IDT) was negative for cefazolin, dexamethasone, fentanyl, methadone, midazolam, propofol, remifentanil, and tranexamic acid (Table 1). Despite negative SPT and IDT, allergy to these medications was not definitively cleared due to the absence of validated predictive data for these tests.

SPT and IDT concentrations (including recommended and actual concentrations) and testing outcomes in this case are detailed in Table 1. The 2023 Anaphylaxis Practice Parameters conditionally recommend that immediate hypersensitivity skin testing (SPT and IDT) or in vitro specific IgE testing be performed on all potential culprit agents a minimum of 4–6 weeks after a POA event [1]. Skin testing should be done with nonirritating concentrations of the drug given the risk of irritant reactions. Currently, the sensitivity, specificity, and predictive values for skin testing to common perioperative agents are poorly defined.

The benefits and risks of drug provocation testing (DPT) were reviewed with the patient, and he elected to proceed with DPT under close medical observation. Medications were selected for the challenge after a multidisciplinary discussion with surgery and anesthesia teams based on the availability of alternative medications. The patient was admitted to the ICU for DPT to IV dexamethasone, IV fentanyl, oral midazolam, IV methadone, and IV tranexamic acid, as well as topical challenges to chlorhexidine, iodine, and tincture of benzoin. For DPT to each IV and oral medication, the patient received 10% of his weight-based dose and was observed for 30 min. If he was asymptomatic, he received the remainder of his dose and was observed for an additional 60 min. Topical challenges were performed by applying the medication to 10% of the estimated body surface area (BSA) needed during surgical prep and observing for 30 min. If he was asymptomatic, the remaining (90%) BSA was covered with medication, with observation for an additional 60 min. The patient tolerated DPT to IV dexamethasone, IV fentanyl, oral midazolam, IV methadone, and IV tranexamic acid, as well as topical challenges to chlorhexidine, iodine, and tincture of benzoin. DPT to cefazolin, propofol, and remifentanil was not performed due to time constraints and/or the nature of medications. The patient was subsequently instructed to avoid cefazolin, propofol, and remifentanil, and these medications were kept as allergies in his medical chart. Information regarding his allergy evaluation was distributed to anesthesiology, orthopedics, and his primary care physician. He subsequently underwent a successful and safe spinal surgery, which included the use of the following medications cleared by his drug allergy evaluation: fentanyl, midazolam, tranexamic acid, chlorhexidine, and iodine. Given the proximity of receiving cefazolin to the patient's index reaction and the relative prevalence of antibiotic allergy in the population compared to propofol and remifentanil, we remain most suspicious of IgE-mediated allergy to cefazolin.

DPT concentrations and testing outcomes for this case are detailed in Table 2. The 2023 Anaphylaxis Practice Parameter conditionally recommends DPT to potential culprit drug agents with negative skin testing. For agents with positive skin testing, DPT is not recommended if alternative agents are available. DPT should also be considered for alternative agents with a risk of cross-reactivity to the suspected culprit agent [1]. Barriers to DPT in POA include a higher risk of severe anaphylaxis due to the route of administration and pharmacologic effects of the drug. There are no established protocols for DPT to most perioperative agents, and few centers perform these high-risk challenges. Some centers have been successful in performing DPT to perioperative agents through collaborations between allergists, anesthesiologists, surgeons, and intensivists. DPT in these centers takes place in locations with high-level monitoring (e.g., ICU, operating room, and anesthesia). Recommended precautions include continuous monitoring and readily available emergency equipment (e.g., oxygen, suction, equipment for intubation, and noninvasive positive pressure ventilation) [11].

While the patient's drug allergy evaluation did not identify a single causative agent, it successfully eliminated eight potential causes of POA, allowing him to proceed with surgery. Limitations of this report include SPT and IDT at concentrations different from the most recent guidelines, specifically for cefazolin. The concentrations used in this case adhered to the recommended guidelines available at the time of testing. This underscores the importance of developing standardized protocols and validating predictive values for skin testing to common perioperative agents. Another limitation was the inability to perform DPT to cefazolin and remifentanil due to time restraints in the critical care setting. Other medications were prioritized over cefazolin and remifentanil due to the higher availability of alternatives. This limitation should be considered with DPT as multiple medications often need to be challenged over multiple hours in a critical care space. Prioritization of agents based on importance and safety should be considered when performing DPT on multiple agents.

In summary, this case demonstrates a systematic approach and highlights challenges for high-risk drug allergy evaluation facilitated by collaboration among allergy, critical care, anesthesiology, and surgery. While cefazolin was the most likely culprit agent given the timing of administration and incidence of cefazolin allergy in the United States, it was essential to evaluate all suspected drugs. Following comprehensive SPT and IDT, further drug allergy testing was performed with DPT in the ICU setting given the high-risk nature of the evaluation. This evaluation ultimately ruled out eight potential POA culprits, allowing the patient to proceed with surgery safely. Performing DPT to potential culprits in POA should be considered if the benefits of the challenge outweigh the risks. Further research is needed to develop standardized SPT, IDT, and DPT protocols to common perioperative agents.

## Figures and Tables

**Table 1:** Recommended and actual skin testing concentrations and test results for the potential culprit drug agents [1, 10].

**Medication**	**Recommended SPT concentration**	**SPT concentration used**	**Recommended IDT concentration**	**IDT concentration used**	**SPT and IDT result**
IV or oral medications					

Cefazolin	20 mg/mL	8 mg/mL	22 mg/mL	8 mg/mL	Negative
	33 mg/mL	25 mg/mL	33 mg/mL	25 mg/mL	
Dexamethasone	4 mg/mL	4 mg/mL	0.004 mg/mL	0.004 mg/mL	Negative
			0.04 mg/mL	0.04 mg/mL	
			0.4 mg/mL	0.4 mg/mL	
			4 mg/mL	4 mg/mL	
Fentanyl	0.05 mg/mL	0.05 mg/mL	0.005 mg/mL	0.005 mg/mL	Negative
Methadone	Not available	10 mg/mL	Not available	0.01 mg/mL	Negative
		1 mg/mL		0.1 mg/mL	
				1 mg/mL	
Midazolam	5 mg/mL	5 mg/mL	0.05 mg/mL	0.5 mg/mL	Negative
Propofol	10 mg/mL	10 mg/mL	1 mg/mL	1 mg/mL	Negative
Remifentanil	0.05 mg/mL	0.05 mg/mL	0.005 mg/mL	0.005 mg/mL	Negative
Tranexamic acid	Undiluted	100 mg/mL	1/10	0.01 mg/mL	Negative
				0.1 mg/mL	

Topical medications					

Chlorhexidine 2%	5 mg/mL	Prick to prick	0.002 mg/mL	Not performed	Negative
Iodine	100 mg/mL	Not performed	Not recommended	Not performed	—
Tincture of benzoin	Not available	Not performed	Not available	Not performed	—

*Note:* Recommended SPT and IDT concentrations were obtained from the 2023 Anaphylaxis Practice Parameter. SPT and IDT concentrations used were informed by available published data on nonirritating concentrations at the time of the procedure. The 2023 Anaphylaxis Practice Parameter was not published at this time.

**Table 2:** DPT methods used and results for the potential culprit drug agents.

**Medication**	**Route of administration**	**Initial dose**	**Second dose**	**Total dose**	**DPT result**
Dexamethasone	IV	1 mg	3 mg	4 mg (0.1 mg/kg)	Tolerated
Fentanyl	IV	2 *μ*g	20 *μ*g	22 *μ*g (0.5 *μ*g/kg)	Tolerated
Methadone	IV	0.1 mg	0.3 mg	0.4 mg (0.01 mg/kg)	Tolerated
Midazolam	Oral	0.5 mg	4.5 mg	5 mg (0.11 mg/kg)	Tolerated
Tranexamic acid	IV	50 mg	400 mg	450 mg (10 mg/kg)	Tolerated
Chlorhexidine 2%	Topical	10% BSA	90% BSA	100% BSA	Tolerated
Iodine	Topical	10% BSA	90% BSA	100% BSA	Tolerated
Tincture of benzoin	Topical	10% BSA	90% BSA	100% BSA	Tolerated

*Note:* Concentrations were informed by published data on nonirritating concentrations for skin testing. Challenges to IV and oral medications were performed using 10% then 90% of the patient's weight-based dose. For topical medications, the total body surface area (BSA) required for a surgical procedure was estimated by a surgeon.

## Data Availability

Data sharing is not applicable to this article as no new data were created or analyzed in this study.

## Nomenclature

BSA: body surface area
DPT: drug provocation testing
ICU: intensive care unit
IDT: intradermal testing
IV: intravenous
NMBA: neuromuscular blocking agent
POA: perioperative anaphylaxis
SPT: skin prick testing
